# Difference imaging from single measurements in diffuse optical tomography: a deep learning approach

**DOI:** 10.1117/1.JBO.27.8.086003

**Published:** 2022-08-25

**Authors:** Shuying Li, Menghao Zhang, Minghao Xue, Quing Zhu

**Affiliations:** a Washington University in St. Louis, Optical and Ultrasound Imaging Lab, Department of Biomedical Engineering, St. Louis, Missouri, United States; b Washington University in St. Louis, Optical and Ultrasound Imaging Lab, Department of Electrical and Systems Engineering, St. Louis, Missouri, United States; c Washington University School of Medicine, Department of Radiology, St. Louis, Missouri, United States

**Keywords:** artificial neural network, breast cancer, deep learning, diffuse optical tomography

## Abstract

**Significance:**

“Difference imaging,” which reconstructs target optical properties using measurements with and without target information, is often used in diffuse optical tomography (DOT) *in vivo* imaging. However, taking additional reference measurements is time consuming, and mismatches between the target medium and the reference medium can cause inaccurate reconstruction.

**Aim:**

We aim to streamline the data acquisition and mitigate the mismatch problems in DOT difference imaging using a deep learning-based approach to generate data from target measurements only.

**Approach:**

We train an artificial neural network to output data for difference imaging from target measurements only. The model is trained and validated on simulation data and tested with simulations, phantom experiments, and clinical data from 56 patients with breast lesions.

**Results:**

The proposed method has comparable performance to the traditional approach using measurements without mismatch between the target side and the reference side, and it outperforms the traditional approach using measurements when there is a mismatch. It also improves the target-to-artifact ratio and lesion localization in patient data.

**Conclusions:**

The proposed method can simplify the data acquisition procedure, mitigate mismatch problems, and improve reconstructed image quality in DOT difference imaging.

## 1 Introduction

Near-infrared diffuse optical tomography (DOT) has shown success in breast cancer diagnosis and treatment response monitoring.^1^^–^^6^ To reduce the ill-posed nature of the DOT inverse problem, prior information from other imaging modalities, such as magnetic resonance imaging (MRI),^2^^,^^7^ x-ray CT or mammography,^8^^,^^9^ and ultrasound (US),^10^^–^^16^ has been employed to improve DOT reconstruction accuracy.

Absolute imaging in DOT uses a single set of measurements to reconstruct spatially distributed absorption and scattering coefficients. However, absolute imaging is sensitive to mismatches between the experimental data and the forward model for several reasons, such as inaccurately known object geometry or uncertain optode coupling coefficients.^17^ Thus, “difference imaging,” which reconstructs target optical properties using measurements with and without the target, is often used in DOT *in-vivo* imaging. Difference imaging can partially cancel out modeling errors that are invariant between the measurements. Typically, the image reconstruction is carried out using the difference between the measurements and a linearized approximation of the forward model.^9^^,^^18^^,^^19^ For example, in clinical breast patient studies, our group normalized the measured diffuse reflectance from the lesion side breast to the contralateral normal breast (the reference side) to produce a “perturbation” for imaging reconstruction.^20^ In practice, a sequence of reference measurements is acquired, and then, according to the chest wall shown in the coregistered US images and the perturbation, the best reference is manually selected for reconstruction. These procedures can significantly increase the data acquisition and postprocessing time. On the other hand, the assumption made in difference imaging that the background properties of the lesion side and reference side are the same may be inaccurate in some cases in clinical studies, and such mismatch can cause incorrect estimation of the lesion’s optical properties and produce image artifacts.^21^^–^^23^

Here, to simplify the data acquisition procedure and to avoid mismatch problems in DOT difference imaging, we propose a new approach using a deep artificial neural network (ANN) to generate perturbation from target measurements only. The ANN model is trained and validated on simulation data, including data generated with the realistic VICTRE breast phantom^24^ and VICTRE breastMass software.^25^ The performance of the model is then tested with simulations, phantom experiments, and patient data.

## 2 Methods

### 2.1 DOT System

A frequency-domain US-guided DOT method and system were used for simulations, phantom experiments, and patient data. The system was previously described in Ref. 26. Briefly, the light source consists of four laser diodes (with wavelengths of 730, 785, 808, and 830 nm) modulated at 140 MHz. The detection of diffuse reflectance is achieved by 14 parallel photomultiplier tube detectors. A local oscillator at 140.02 MHz is used to demodulate the detected signals to 20 kHz. Then the signals are digitized and sampled by two eight-channel A/D data acquisition cards. A US transducer in the middle of the DOT probe provides the coreregistered B-scan US images.

### 2.2 Dataset

#### 2.2.1 Simulation

A total of 43,398 sets of measurements from finite element method (FEM) and Monte Carlo simulations^27^ were used: 80% were randomly chosen as the training set and the rest were used as the validation set. The training set was used to fit the model, and the validation set was used to determine the ANN structure and to select proper hyperparameters. Different target sizes, depths, shapes, and absorption and reduced scattering coefficients ($\mu_{a}$ and ${\mu_{s}}^{\prime }$, respectively) were considered. Tissue heterogeneity was simulated with realistic digital breasts.^24^ The target and background properties of the training and validation dataset are given in Table 1. More details about the setup of the simulations can be found in Refs. 28 and 29.

**Table 1:** Target, tissue, and chest wall properties in the training and validation set.

	FEM	Monte Carlo
Target shape	Spherical	Spherical, irregular^25^
Target radius (cm)^a^	0.5 to 1.5	0.375 to 1.5
Target center depth (cm)	0.6 to 2.5	0.7 to 2.7
Target $\mu_{a}$ (${\mathrm{cm}}^{-1}$)	0.08 to 0.30	0.10 to 0.30
Target ${\mu_{s}}^{\prime }$ (${\mathrm{cm}}^{-1}$)	4 to 8	7
Tissue $\mu_{a}$ (${\mathrm{cm}}^{-1}$)	0.01 to 0.06	Digital breasts with 20% to 80% fat;^24^ no chest wall
Tissue ${\mu_{s}}^{\prime }$ (${\mathrm{cm}}^{-1}$)	4 to 8	
Chest wall depth (cm)	1.6 to 5	
Chest wall tilting angle (deg)	−10 to 10	
Chest wall $\mu_{a}$ (${\mathrm{cm}}^{-1}$)	0.1 to 0.2	
Chest wall ${\mu_{s}}^{\prime }$ (${\mathrm{cm}}^{-1}$)	4 to 8	

a For nonspherical targets, the radii are calculated as half of the largest dimensions in the z direction.

To demonstrate the generalization of the model, we generated 180 additional sets of simulation data for testing. This dataset covered various target sizes, depths, and absorption coefficients, and we made sure that the $\mu_{a}$ and ${\mu_{s}}^{\prime }$ of targets, the breast tissue, and the chest wall in this dataset were different from that of the training and validation sets.

#### 2.2.2 Phantom experiments

To further evaluate the performance of the model, phantom experiments and patient data were used as the test set because they are more representative of future unseen clinical data. Two sets of solid targets with high contrast ($\mu_{a}=0.23\;{\mathrm{cm}}^{-1}$) and low contrast ($\mu_{a}=0.11\;{\mathrm{cm}}^{-1}$) were placed in Intralipid^®^ solution with a $\mu_{a}$ of $0.015\;{\mathrm{cm}}^{-1}$ and a ${\mu_{s}}^{\prime }$ of $7.3\;{\mathrm{cm}}^{-1}$, both measured at 730 nm.^26^ These targets had diameters of 1, 2, and 3 cm and were placed at different depths (central depths of 1.5, 2, and 2.5 cm).

To demonstrate the advantage of the proposed method in avoiding reconstruction inaccuracy due to mismatch between the target and reference measurements, we also conducted phantom experiments with chest wall mismatch. A gelatin-intralipid phantom^30^ with a $\mu_{a}$ of $0.076\;{\mathrm{cm}}^{-1}$ and a ${\mu_{s}}^{\prime }$ of $9.8\;{\mathrm{cm}}^{-1}$ measured at 730 nm was used to simulate the chest wall. Chest wall mismatch was created by placing the chest wall deeper at the target side than the reference side.

#### 2.2.3 Clinical data

Patient data from 43 benign (22 fibroadenomas or proliferative lesions (PL) and 21 low-risk benign lesions of fibrocystic changes or complex cysts) and 13 malignant cases were used to evaluate the performance of the proposed method. This group of 56 patients had an average age of 49.2 years (±13.8 years). The lesion radii in the z-direction ranged from 0.5 to 1.5 cm. The study was approved by the local IRB and was HIPAA compliant. Written informed consent was obtained from all patients. The data used in this paper were deidentified.

### 2.3 Artificial Neural Network

In traditional difference imaging for US-guided DOT, the normalized perturbation, pert, was calculated from the frequency-domain measurements of the lesion-side breast normalized to the contralateral reference-side breast:

$$\text{pert}=\frac{U_{l}-U_{r}}{U_{r}}=(\frac{A_{l}}{A_{r}}\;\cos(\phi_{l}-\phi_{r})-1)+j\frac{A_{l}}{A_{r}}\;\sin(\phi_{l}-\phi_{r}),$$ (1)

where $U_{l}$ and $U_{r}$ are the lesion-side and the reference-side measurements, respectively. Under the assumption that the only difference between the lesion side and the reference side is that the lesion has a higher absorption coefficient than the background, $A_{l}$ should be smaller than $A_{r}$. Simulations have shown that the phase difference between the two sides $\left|\phi_{l}-\phi_{r}\right|$ does exceed 90 deg;^31^ thus the real part of the normalized perturbation should be between −1 and 0.

Multilayer perceptron (MLP), a type of feedforward ANN, can approximate any continuous mapping function from one finite-dimensional space to another,^32^ and this mapping function can be learned using a data-driven approach. Hence, we trained an MLP-based ANN to output perturbation measurements *pert* from target measurements $U_{l}$ only. The nonlinear $U_{l}$-to-*pert* mapping function, or the weight of the ANN, was learned from simulations with known $U_{l}$ and *pert*.

**Fig. 1:**
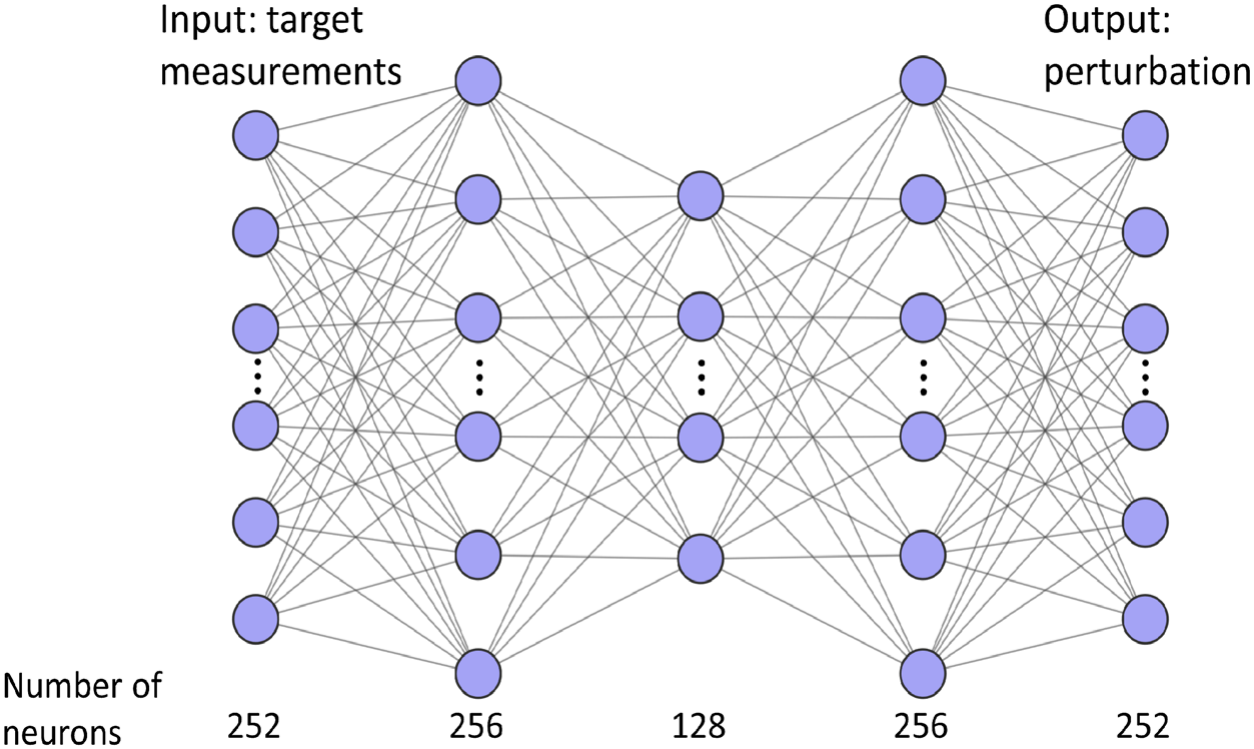
ANN structure.

As shown in Fig. 1, the input and output layers of the ANN both have 252 neurons, and the three hidden layers have 256, 128, and 256 neurons, respectively. To introduce nonlinearity to the neural network, a rectified linear unit activation function was used after each fully connected layer except the last one. The inputs of the network are the lesion side log amplitude, $\log(A_{l}\rho^{2})$, and phase, $\phi_{r}$, of simulated light reflectance on the lesion side with 2% random Gaussian noise added. Here, $A_{l}$ is the amplitude and ρ is the source–detector distance. The outputs are the real and imaginary parts of the normalized perturbation. To avoid scaling problems for different datasets, the $\log(A_{l}\rho^{2})$ values were variously offset in each set of measurements to keep the maximums of $\log(A_{l}\rho^{2})$ the same. Similarly, the minimums of $\phi_{r}$ were kept the same. The output of the ANN is the real and imaginary parts of the normalized perturbation. The mean square error was used as the loss function. The network was implemented in PyTorch^33^ and was trained for 200 epochs with a batch size of 64, using the Adam solver^34^ with a learning rate of 1e-4 and a weight decay of 1e-5. Validation loss was monitored to avoid overfitting. The learning rate was reduced by a factor of 0.2 of its previous value when the validation loss did not decrease for three epochs. The training procedure took ∼20  min on an NVIDIA Quadro P2000, and the testing procedure took <0.1  s to generate a perturbation from one set of target measurements.

### 2.4 Image Reconstruction and Quantification

The DOT inverse problem was linearized using the Born approximation and was formulated as the following regularized optimization problem:^35^

$$f(x)=\arg\;\min_{\delta \mu_{a}}({\left\|\text{pert}-W\delta \mu_{a}\right\|}^{2}+\frac{\lambda }{2}{\left\|\delta \mu_{a}\right\|}^{2}).$$ (2)

Here, $\delta \mu_{a}$ represents the unknown changes in target $\mu_{a}$ compared with the reference side, W is a sensitivity matrix calculated using the Born approximation, and λ is a regularization parameter. The conjugate gradient algorithm and a dual mesh scheme using coregistered US guidance were employed to solve the inverse problem.^36^ For patient data, the total hemoglobin (tHb) distribution was calculated using the reconstructed absorption maps of all four wavelengths.

For phantom data, the structural similarity index (SSIM) was used to evaluate the similarity between the reconstructed images and the ground truths. To evaluate the reconstructed image quality of patient data that did not have ground truths, we used a semiautomated CNN to segment lesions from the coregistered US images of the patient data.^37^^,^^38^ After each lesion was segmented, its x- and z-centroids were calculated (assuming the y-centroid to be 0), and the distance between the centroid and the reconstructed lesion center of mass was calculated. For each depth in the reconstructed image, we defined the lesion area to be three times the US-segmented width from the x/y-centroid, defined outside to be the artifact area, and then we calculated $\max({\mathrm{tHb}}_{\text{target}})/\max({\mathrm{tHb}}_{\text{artifact}})$.

The Wilcoxon rank sum test was used to evaluate the statistical significance, with a p-value of <0.05 being considered statistically significant. All image reconstruction and statistical tests were conducted using MATLAB 2021a.

## 3 Results

### 3.1 Testing on Simulation Data

**Fig. 2:**
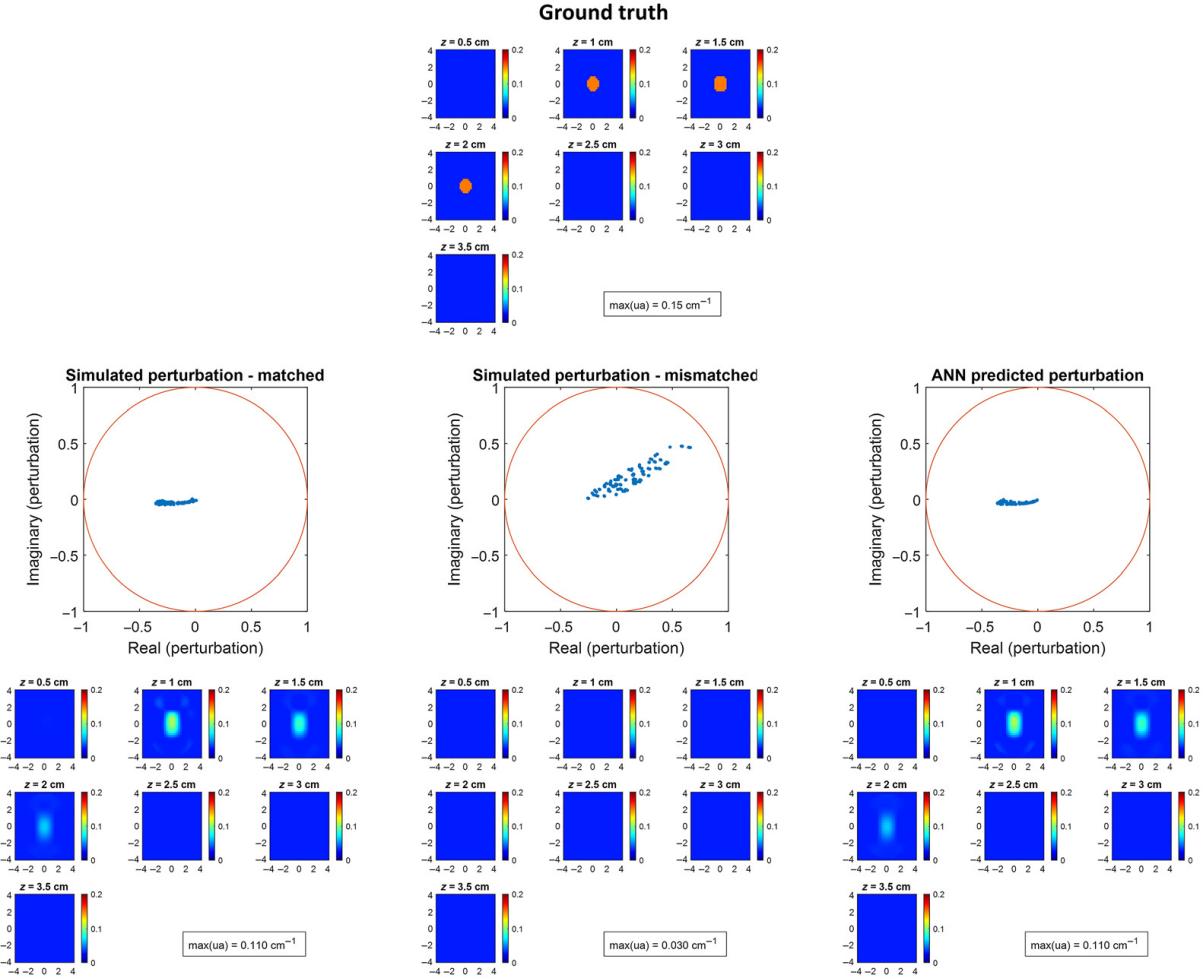
Ground truth, simulated perturbation without chest wall mismatch (middle row, left), simulated perturbation with chest wall mismatch (middle row, middle), ANN-predicted perturbation (middle row, right), and their corresponding reconstructed images (bottom row). The target has $\mu_{a}=0.15\;{\mathrm{cm}}^{-1}$, radius=1  cm, and depth=1.5  cm. Chest wall depth is 3 cm on the target side and the matched reference side and is 2 cm on the mismatched reference side.

For the 180 sets of simulation data in the test set, the averaged absolute difference between the reconstructed maximum $\mu_{a}$ from ANN predicted perturbation and from matched perturbation was $0.0068\;{\mathrm{cm}}^{-1}$ (95% CI: 0.0062 to $0.0074\;{\mathrm{cm}}^{-1}$). Figure 2 shows one ground truth, simulated perturbation without mismatch, simulated perturbation with chest wall mismatch, ANN-predicted perturbation, and their corresponding reconstructed images. When there is no mismatch, the real part of the measured perturbation is negative and localized. When the reference side chest wall is shallower than the target side chest wall, both the real and imaginary parts of the perturbation move in the positive direction, and the target is not reconstructed. The ANN-predicted perturbation is similar to the perturbation without mismatch, and its corresponding reconstructed image shows a similar target shape, depth profile, and $\mu_{a}$ to the image using perturbation without mismatch.

### 3.2 Testing on Phantom Data

**Fig. 3:**
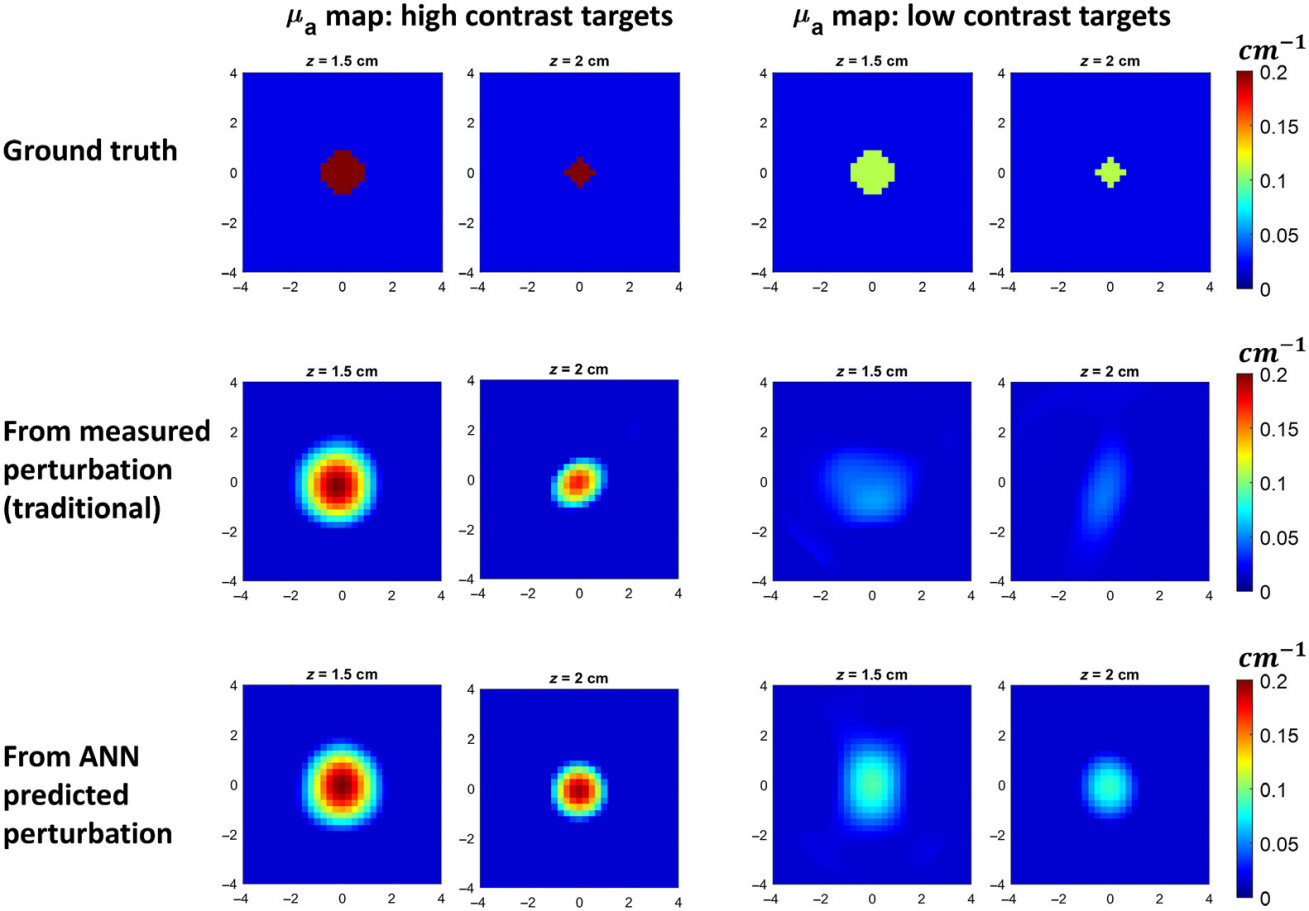
Reconstructed phantom images of high and low contrast targets with different radii at a 2-cm depth. For better visualization, we show only the top layer of the reconstructed image containing the target.

Figure 3 shows images of phantoms reconstructed using the ANN-predicted perturbation and the measured perturbation. For high contrast targets, the two methods give similar reconstructed target sizes, shapes, and $\mu_{a}$ values. However, for low contrast targets, the reconstruction using the measured perturbation has distorted shapes, and the reconstructed $\mu_{a}$ values are lower than the ground truths, whereas the reconstruction using the ANN-predicted perturbation gives shapes and $\mu_{a}$ closer to the ground truths.

**Fig. 4:**
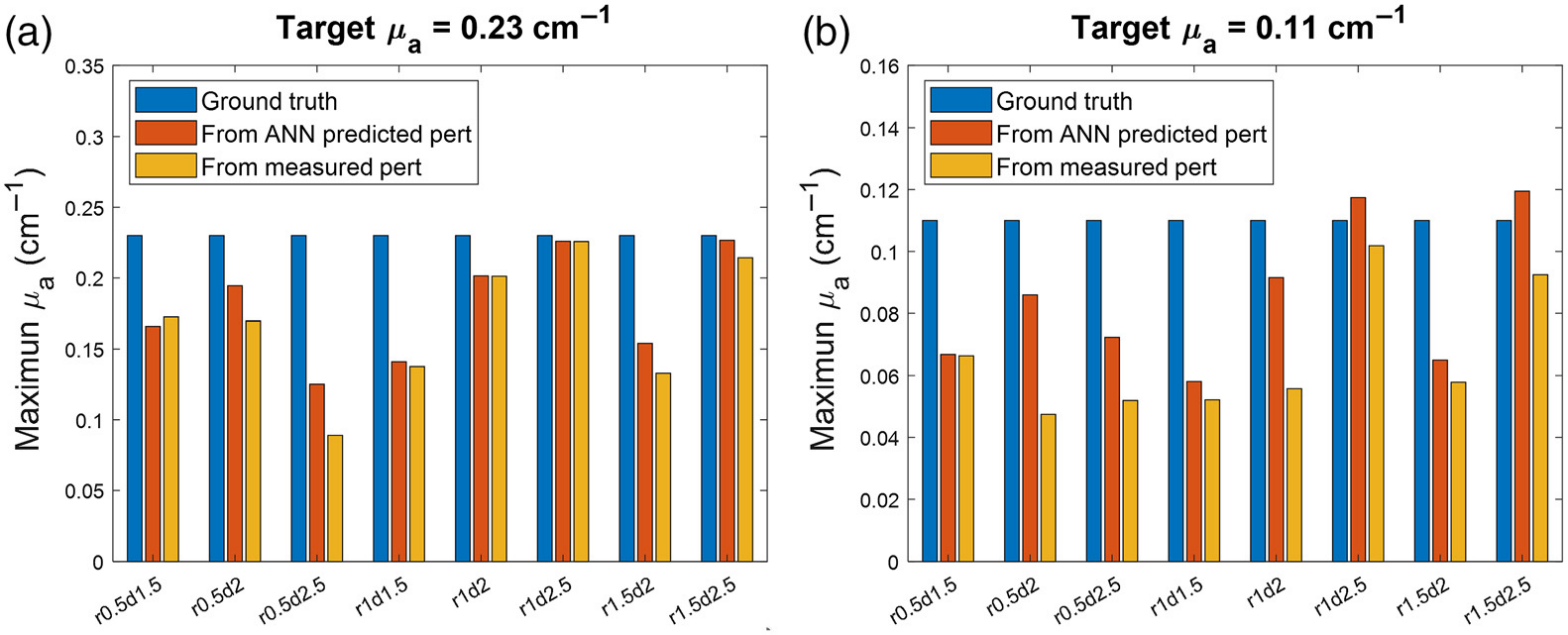
The maximum reconstructed $\mu_{a}$ for (a) high and (b) low contrast targets with different radii (r) and depths (d).

Figure 4 shows the maximum reconstructed $\mu_{a}$ for high and low contrast targets with different radii and depths. In all tested cases, except for the high contrast target with a 0.5-cm radius and a 1.5-cm depth, the ANN-predicted perturbations give the maximum reconstructed $\mu_{a}$ values closer to the ground truth than the measured perturbation does.

**Fig. 5:**
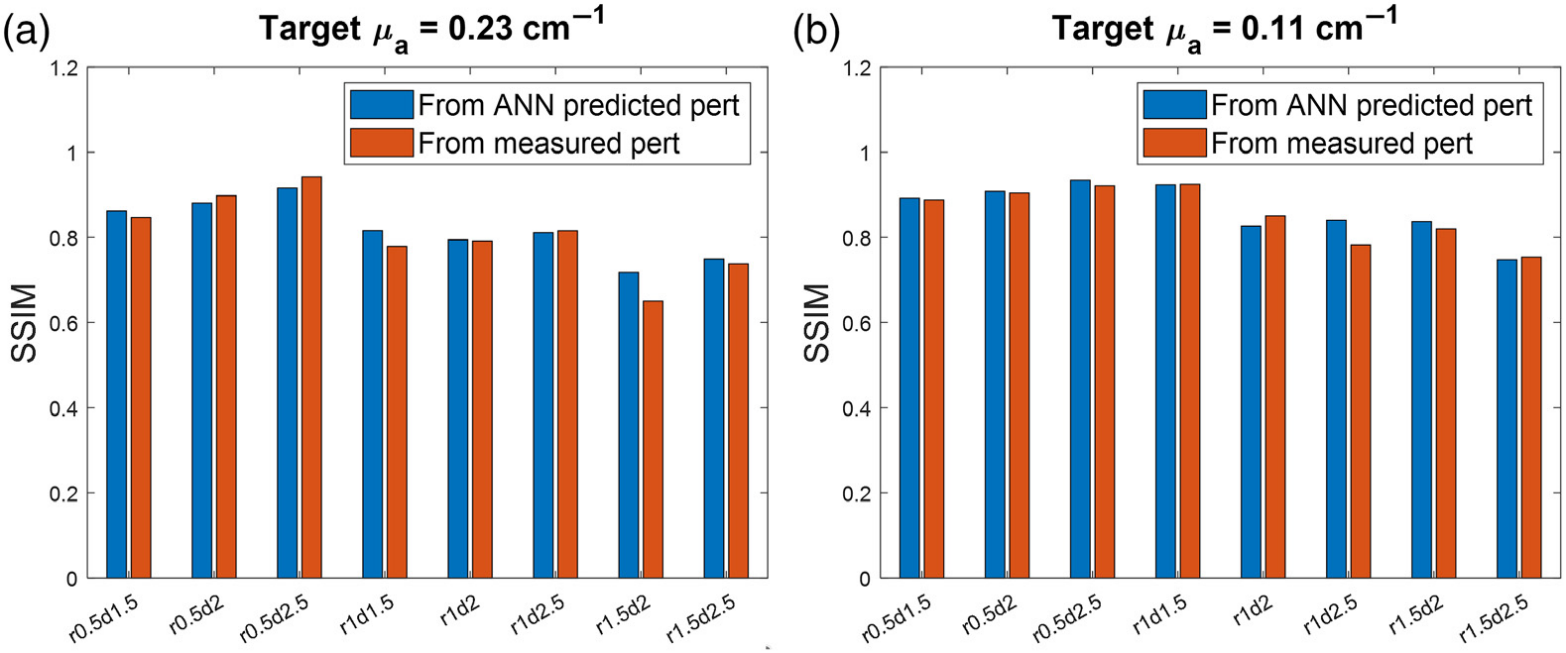
SSIM values between reconstructed images and the ground truths for (a) high and (b) low contrast targets with different radii (r) and depths (d).

Figure 5 shows the SSIM values between the reconstructed images using the corresponding ground truths. For all tested cases, images generated using ANN-predicted perturbations have SSIM values higher than or similar to (difference<3%) those using measured perturbations.

**Fig. 6:**
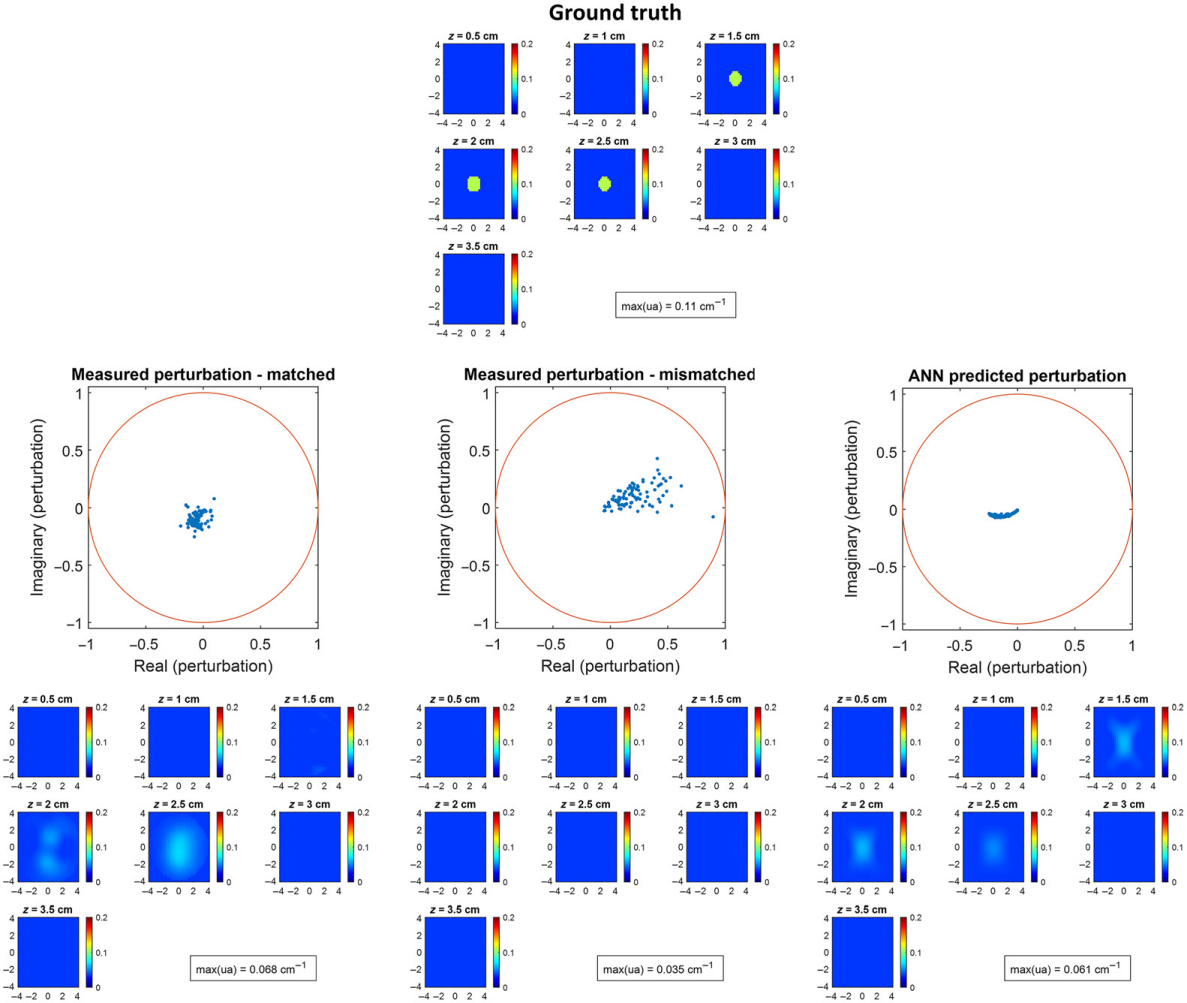
Ground truth for phantom experiment with chest wall (top row), measured perturbation without chest wall mismatch (middle row, left), measured perturbation with chest wall mismatch (middle row, middle), ANN-predicted perturbation (middle row, right), and their corresponding reconstructed images (bottom row). The target has $\mu_{a}=0.11\;{\mathrm{cm}}^{-1}$, radius=1  cm, and depth=2  cm. Chest wall depth is 3 cm on the target side and the matched reference side and is 2 cm on the mismatched reference side.

Figure 6 shows measured perturbations without and with chest wall mismatch and the ANN-predicted perturbation and their corresponding reconstructed images. When there is no chest wall mismatch, the real part of the measured perturbation is negative and localized, with a few outliers. The reconstructed target is mostly located in the bottom layer. When the reference side chest wall is shallower than the target side chest wall, both the real and imaginary parts of the measured perturbation move in the positive direction, and the target is not reconstructed. The ANN-predicted perturbation generated an image that shows the target with three layers, which is closer to the ground truth than the other two.

### 3.3 Testing on Patient Data

**Fig. 7:**
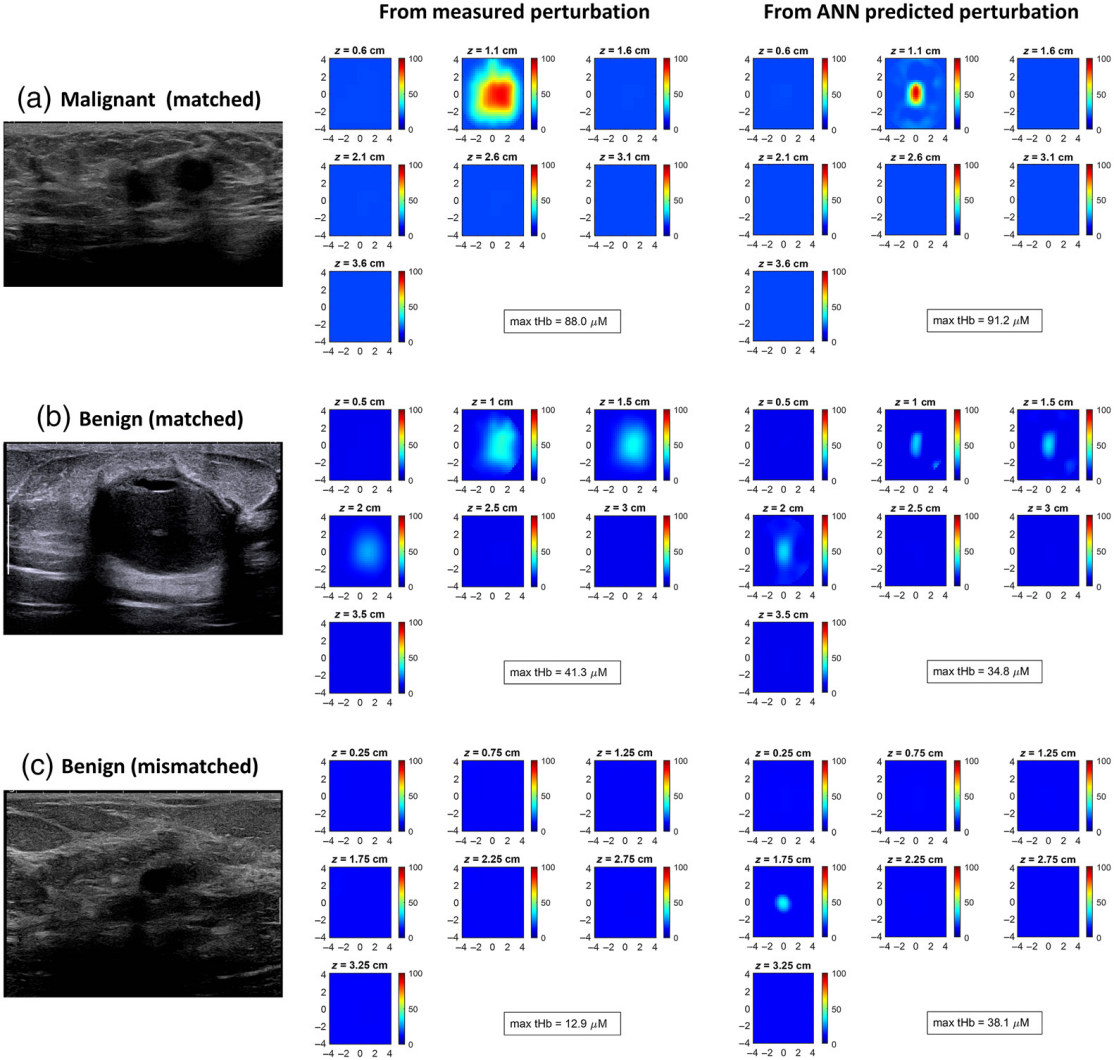
Coregistered US images (left column), tHb maps reconstructed using the measured perturbation (middle column), and tHb maps reconstructed using the ANN-predicted perturbation (right column) for (a) a malignant tumor and (b) and (c) benign lesions. In (c), there is a mismatch between the lesion and reference side measurements. Color bars indicate the tHb level, in units of μM.

**Fig. 8:**
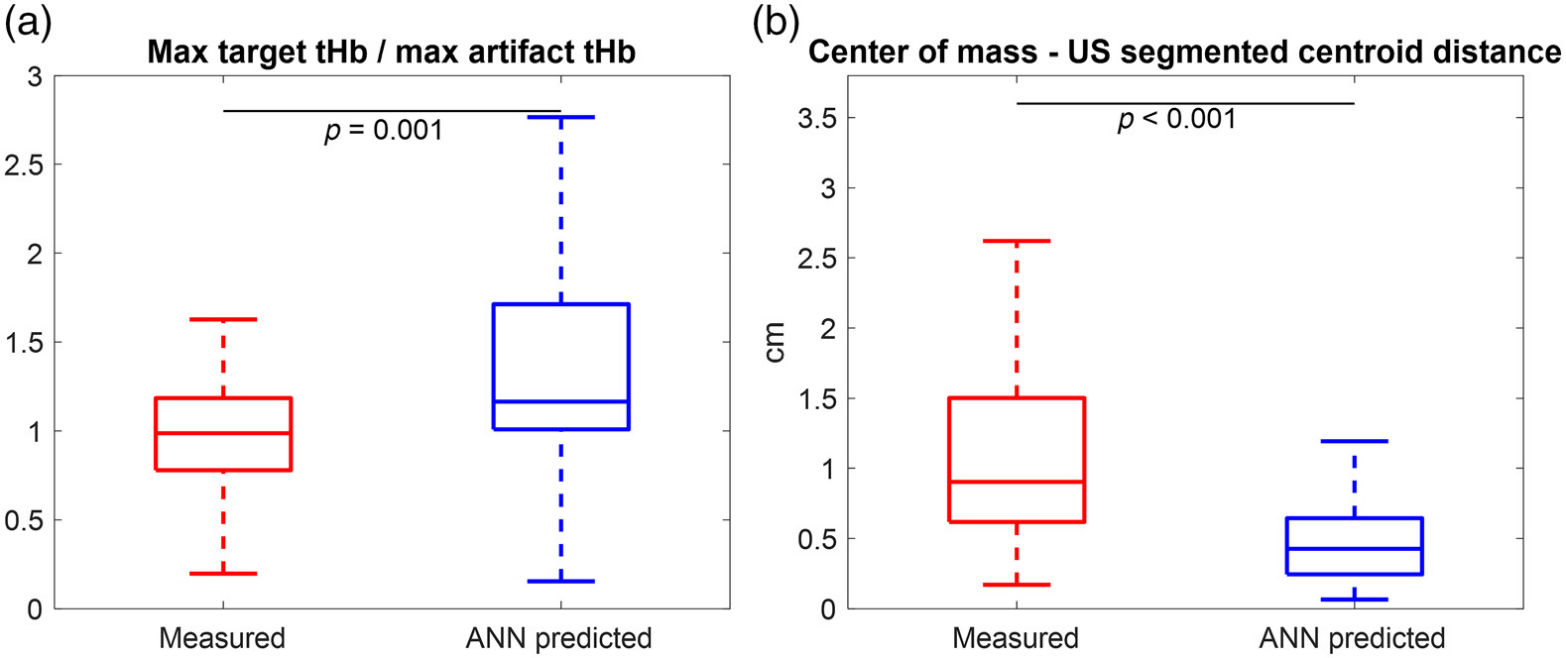
Box plots of (a) the ratio of the maximum values of reconstructed target tHb over artifact tHb and (b) the reconstructed center of mass to US segmented centroid distance for all 56 lesions, using the measured and ANN-predicted perturbations.

Figure 7 shows representative tHb maps from three patients: one with a malignant tumor [Fig. 7(a)] and two with benign lesions [Figs. 7(b) and 7(c)]. To calculate the measured perturbation, the best reference was manually selected according to the chest wall position in the coregistered US image and the perturbation. Figure 7(a) shows the coregistered US image of a 59-year-old patient with invasive ductal carcinoma with a 1.1-cm deep lesion that measures 2.4 and 1.0 cm in the x- and z-directions, respectively. Figure 7(b) shows a 52-year-old patient with a benign cyst that is located 1.5 cm deep and measures 2.8 and 2.0 cm in the x and z directions, respectively. In Figs. 7(a) and 7(b), no significant mismatch was found by evaluating the coregistered US images and the measured perturbations. Compared with the measured perturbation, the ANN-predicted perturbation generated images with similar depth profiles and the maximum absorption coefficients, but they are more focused and centralized. Figure 7(c) shows a 48-year-old patient with a small pseudoangiomatous stromal hyperplasia that measures 1.4 and 0.8 cm in the x and z directions, respectively. By evaluating the measured perturbation, we found that there was a mismatch between the lesion and reference side measurements. In the reconstructed image, the lesion was not properly reconstructed and did not show up. Using the proposed deep learning-based approach, the lesion was successfully reconstructed in the corresponding area of the US image, using the perturbation generated from the target side measurements only. Figures 8(a) and 8(b) show box plots of the ratio of the maximum values of the reconstructed target tHb to the artifact tHb and the reconstructed center of mass to US segmented centroid distances for all 56 cases, respectively. The images reconstructed using the ANN-predicted perturbations have higher target tHb/artifact tHb ratios, and the lesions are closer to the US-segmented lesion positions.

**Fig. 9:**
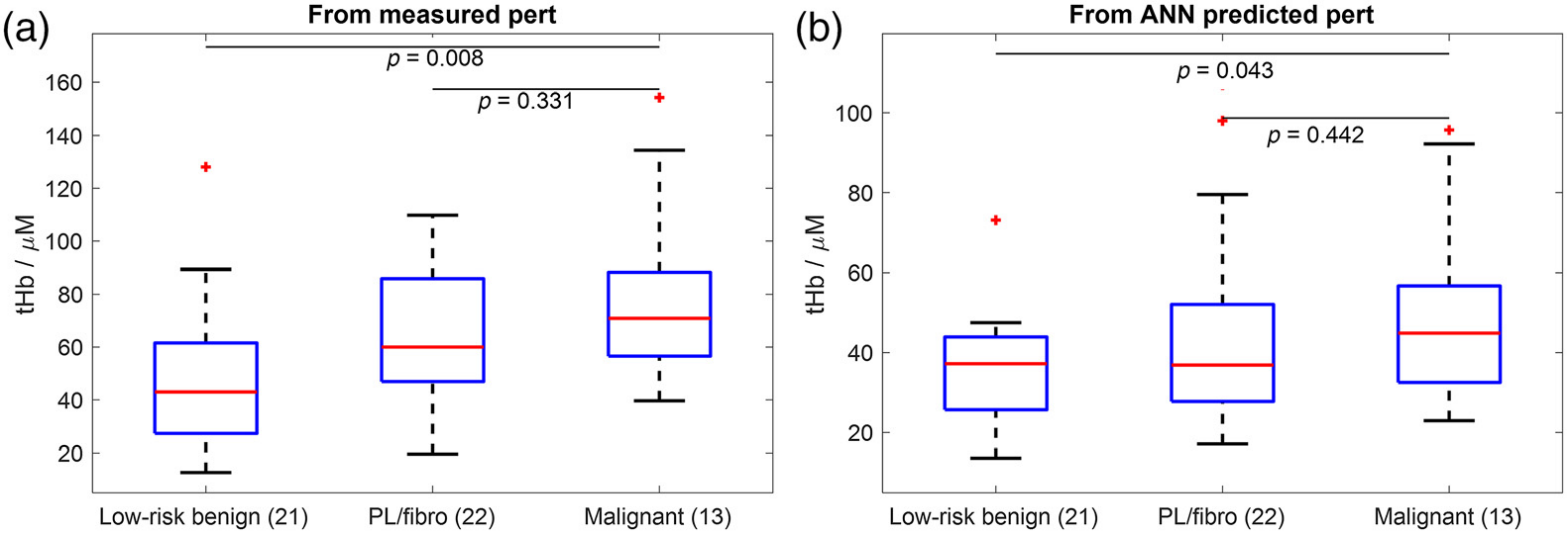
Box plots of the maximum reconstructed tHb for 21 low-risk benign lesions, 22 fibroadenomas (fibro) or PL, and 13 malignant cases, using (a) measured perturbations and (b) ANN-predicted perturbations.

Figure 9 shows box plots of the maximum tHb for 21 low-risk benign lesions, 22 fibroadenomas (fibro) or PL, and 13 malignant cases, reconstructed using the measured and ANN-predicted perturbations. The tHb values calculated for all target files were averaged for each patient. The tHb values reconstructed using the ANN-predicted perturbations are overall lower than those using the measured perturbations. The tHb maps of low-risk benign and malignant lesions reconstructed from the ANN-predicted perturbations are significantly different, although the p-value is slightly higher than those reconstructed from the measured perturbations calculated using the best manually selected references. For reconstructions using ANN-predicted perturbations, the PL/fibro group has larger variations than the other two groups.

## 4 Discussion

We designed an ANN to generate data for DOT difference imaging from target measurements only. The ANN was trained and validated using simulation data that included a wide range of realistic background tissues as well as target sizes, shapes, depths, and optical properties to account for individual differences in patients. To simulate the noise in actual measurements, we included Monte Carlo simulations that contain photon noise and added Gaussian noise to the simulated target measurements. The model was then tested with phantom and clinical data. It performed comparably to the approach using measurements without mismatch, and it outperformed the approach using measurements when there was a mismatch between the target and reference measurements. This is because the ground truths for training the ANN are the perturbations calculated from matched target and reference measurements. Hence, unlike the traditional approach in which the perturbation highly depends on the reference measurements, the ANN will always output a matched perturbation. This predictive approach can simplify data acquisition and mitigate mismatch problems in DOT difference imaging. It also improved the image quality in terms of the target-to-artifact-ratio and lesion localization.

This study has several limitations. First, the simulations included in the training set are limited. The proposed approach can be further improved using more complex simulations, such as larger lesions with peripheral Hb distribution, off-center lesions, and complex chest walls. Also, because perturbations without system noise were not available, we do not have ground truths for the phantom experiments, so we cannot fine-tune the ANN model with phantom experiments. However, when testing the trained model on phantom data, most of the images reconstructed using ANN-predicted perturbations were closer to the ground truth than those using true measurements without mismatch, indicating a good generalization of the model on experimental data. For patient data, the tHb values reconstructed using ANN-predicted perturbation are overall lower than those using measured perturbation; thus, a new and lower threshold for separating benign from malignant lesions must be set. The performance of the ANN is better than or comparable to that of the measured perturbation, regardless of target sizes, for phantom data (Figs. 4 and 5). Nevertheless, in clinical data, we found that the ANN performs better than the measurements in distinguishing smaller benign and malignant lesions with z dimensions ≤1 cm but worse in distinguishing larger lesions. This relatively poor performance likely reflects the fact that larger lesion produced perturbations are more difficult to completely separate from the tissue background. In addition, the ANN performance for 808 and 830 nm is worse than 730 and 785 nm, which may be related to the sensitivity of these two wavelengths to the chest wall. For a prospective clinical application, we would streamline the reconstruction based on lesion size measured by US and decide if the ANN model or measured perturbation would be used for reconstruction. Finally, for other DOT systems with different configurations, the ANN may be modified according to the specific DOT systems and retrained with the new data.

## Code, Data, and Materials Availability

The associated code has been uploaded to Code Ocean, https://codeocean.com/capsule/1476758/tree. Data are available from the corresponding author upon reasonable request.
